# Formes hémorragiques graves de la fièvre de la vallée du Rift: à propos de 5 cas

**DOI:** 10.11604/pamj.2016.24.73.9573

**Published:** 2016-05-24

**Authors:** Mohamed Lemine Ould Salem, Sidi El Wafi Ould Baba, Fatimetou Zahra Fall-Malick, Boushab Mohamed Boushab, Sidi Mohamed Ghaber, Abdelwedoud Mokhtar

**Affiliations:** 1 Service des Laboratoires, Centre Hospitalier National de Nouakchott, Mauritanie; 2 Faculté de Médecine de Nouakchott, Mauritanie; 3 Service de Médecine interne, Centre Hospitalier National de Nouakchott, Mauritanie; 4 Institut National d'Hépato-virologie de Nouakchott, Mauritanie; 5 Service de Médecine interne du Centre Hospitalier d'Aïoun, Mauritanie

**Keywords:** Fièvre de la Vallée du Rift, syndrome hémorragique, Mauritanie, Rift Valley fever, hemorrhagic syndrome, Mauritania

## Abstract

La fièvre de la vallée du Rift (FVR) est une arbovirose due à un virus à ARN appartenant à la famille de Bunyaviridae (genre phlebovirus). C'est une zoonose touchant principalement les animaux mais pouvant aussi contaminer l'homme, soit directement par la manipulation des viandes ou avortons d'animaux malades ou indirectement par la piqure de moustiques infectées (*Aèdes sp, anophèles sp, Culex sp*). Dans la majorité des cas, l'infection humaine à FVR est asymptomatique, mais elle peut également se manifester par un syndrome fébrile modérée d’évolution favorable. Néanmoins, certains patients peuvent développer un syndrome hémorragique et/ou des lésions neurologiques d’évolution mortelle. Nous décrivons l’évolution de cinq cas de patients atteints de la FVR, admis dans le service de médecine interne du Centre Hospitalier National de Nouakchott (Mauritanie), le mois d'Octobre 2015 et présentant tous, un syndrome hémorragique dans un contexte fébrile. L’évolution n’était favorable que pour 2 des cinq patients. Les 3 autres sont décédés, deux dans un tableau de choc hémorragique et dans un état de choc septique.

## Abstract (en)

Rift Valley fever (RVF) is an arbovirus caused by an RNA virus belonging to family Bunyaviridae (genus phlebovirus). It is a zoonosis that primarily affects animals but it also has the capacity to infect humans, either by handling meat, runts of sick animals or, indirectly, by the bite of infected mosquitoes (Aedes sp, Anopheles sp, Culex sp). In most cases, RVF infection in humans is asymptomatic, but it can also manifest as moderate febrile syndrome with a favorable outcome. However, some patients may develop hemorrhagic syndrome and/or neurological damages with a fatal evolution. We present a case study of the development of 5 patients with RVF associated with hemorrhagic fever syndrome admitted to the internal medicine department at National Hospital Center in Nouakchott (Mauritania), in October 2015. The outcome was favorable for two of the five patients. The other 3 died, two of hemorrhagic shock and one of septic shock.

## Introduction

La fièvre de la vallée du Rift (FVR) est une arbovirose due à un virus à ARN appartenant à la famille de*Bunyaviridae* (genre *phlebovirus*) touchant principalement les animaux, mais pouvant aussi contaminer l′homme, soit directement par la manipulation des viandes ou avortons d'animaux malades ou indirectement par la piqure de moustiques infectées (*Aèdes sp, anophèles sp, Culex sp*) [1, 2]. Cette zoonose sévit essentiellement en Afrique [1], mais des cas ont été signalés en 2000, en Arabie Saoudite et au Yémen [3, 4]. Chez les ruminants domestiques, elle est caractérisée par des taux élevés d'avortements et de morts, représentant des pertes économiques substantielles. Chez l'Homme, les symptômes sont généralement bénins, mais dans les cas graves, des hémorragies, une méningo-encéphalite, une rétinopathie évoluant vers la mort peuvent survenir [1, 5]. Nous rapportons, cinq cas de formes hémorragiques graves de FVR observés dans le service de médecine interne du Centre Hospitalier National de Nouakchott (Mauritanie). L'objectif de ce travail est de dégager des recommandations de prise en charge précoce des cas et de sensibiliser les autorités aux mesures globales de prévention.

## Patients et observation

### Patient N° 1

SH, âgé de 18 ans éleveur résidant à Tamchekett/Hodh El Gharbi (Sud-Est de la Mauritanie), sans antécédents médico-chirurgicaux notables, admis dans le service de médecine interne du Centre Hospitalier National (CHN) de Nouakchott le 1/10/2015 pour un syndrome hémorragique associant un épistaxis, une gingivorragie et une hématémèse de moyenne abondance. L'examen clinique à l'admission montrait une gingivorragie, une pâleur cutanéo-muqueuse et une polypnée (20 cycle/min) dans un contexte fébrile (39°C). L'hémogramme montrait une anémie à 5,6g/dl, une thrombopénie sévère à 20 000/mm^3^ et une hyperleucocytose à 13 000/mm^3^. On a noté également une perturbation de la fonction rénale et de la fonction hépatique. Le test de diagnostic rapide (TDR) pour le paludisme, l'Antigène Hbs (AgHbs) et la sérologie VIH étaient négatifs. Un prélèvement sanguin a été envoyé à l'Institut National de Recherches en Santé Publique (INRSP) de Nouakchott (Mauritanie) dans le cadre d'une surveillance. Le patient avait bénéficié de transfusions sanguines (10 poches de concentré plaquettaire et 2 de culots globulaires de sang iso-groupe isorhesus) et d'un traitement symptomatique (antipyrétique et solutés). Malgré la prise en charge médicale, son état s'est par la suite aggravé, par survenue d'une hématurie totale et le patient décédait au deuxième jour d'hospitalisation.

### Patient N°2

GI, de sexe masculin, âgé de 36 ans, éleveur, résidant à Maghtaa-Lahjar/Brakna (Centre de la Mauritanie) sans antécédents particuliers notables, admis au service de médecine interne du CHN le 02/10/2015 pour des épistaxis et des céphalées dans un contexte fébrile. L'examen clinique à l'admission objectivait des céphalées intenses, des nausées et un épistaxis dans un contexte fébrile (40°C) L'Hémogramme montrait une hyperleucocytose (18 000/mm^3^), une anémie (9,7g/dl), et une thrombopénie (42 000/mm^3^). Le TDR pour le paludisme était négatif, la C Réactive Protéine (CRP) était élevée (96 mg/l), le bilan hépato-rénal était normal et l'AgHBs était négatif. Un prélèvement a été envoyé à l'INRSP pour la sérologie des fièvres hémorragiques. Les principaux résultats sont notés dans le Tableau 1. Le patient avait bénéficié de transfusions sanguines (cinq concentrés plaquettaires), d'une antibiothérapie et d'un traitement symptomatique. Le patient décédait au huitième jour de son hospitalisation, dans un tableau évoquant un choc septique.

**Tableau 1:** Caractéristiques des cinq patients atteints de la FVR

Paramètres	Patient 1 (SH)	Patient 2 (GI)	Patient 3 (DM)	Patient 4(GA)	Patient 5(AE)
**Age (ans)**	**18**	**36**	**21**	**17**	**29**
**Sexe**	Masculin	Masculin	Masculin	Masculin	Féminin
**Résidence**	Tamchekett/Hodh El Gharbi	Maaghta-Lahjar	Tidjikja	Mederdra	Djiguéni
**Profession**	Eleveur	Eleveur	Eleveur	sans	Sans
**Notion de consommation ou contact avec les produits biologiques d'animaux malades**	Oui	Oui	Oui	Non	Oui
**Délai avant hospitalisation**	3 jours	2 jours	Supérieur à 7 jours	4 jours	6 jours
**Signes cliniques à l'admission**	Fièvre Epistaxis Gingivorragies Hématémèse	Fièvre Epistaxis Céphalées	Fièvre Epistaxis Hématémèse Dyspnée au repos	Fièvre Epistaxis Hématémèse	Fièvre Epistaxis Gingivorragies
**Bilan biologique à l'admission**	GB (13 000/mm^3^) Hb (5,6g/dl) PLT (20 000 mm3) Urée = 17,1mmol/l, Créatininémie =466,4 umol/l Natrémie (135 mmol/l) kaliémie (5,5 mmol/l) CRP 9mg/l)	GB (18 000/mm^3^) Hb (9,7 g/dl) PLT (42 000 /mm3) Urée = 5.5mmol/l, Créatininémie (75umol/l) CRP (96 mg/l) Natrémie (135 mmol/l) Kaliémie (5,5 mmol/l)	GB (1500/mm^3^) Hb (9,5 g/dl) PLT (90 000/mm^3^) Urée 38.5mmol/l) Créatinémie (1038 umol/l) Natrémie (129 mmol/l) kaliémie (5 mmol) ALAT (680 UI/l) ASAT (410 UI/l)	GB (4300/mm^3^) Hb (13,3g/dl) PLT (6600/mm^3^) Urée (3.32mmoll) Créatinémie (79 umol/l) CRP (<6mg/l)	GB = 3200 Hb (8,3 g/dl) PLT (7000/mm^3^), Urée (27.8mmol/l) Créatinémie (380 umol/l) ALAT (1260 UI/l) ASAT (1190UI/l)
**Groupe sanguin rhésus**	AB positif	O positif	O négatif	O positif	A positif
**Diagnostic de la FVR par ELISA et**					
**RT-PCR**	Positif	Positif	Positif	Positif	Positif

GB: globules blancs, Hb: hémoglobine, PLT: plaquettes, ALAT (Alanine mino-transférase), ASAT: (Aspartate amino-transférase), ELISA: Enzyme-linked immunosorbent assay, RT-PCR: reverse-transcriptase polymerase chain reaction

### Patient N° 3

DM, de sexe masculin âgé de 21 ans, originaire de Tagant (Centre du pays), éleveur sans antécédents pathologiques notables, admis dans le service de médecine interne du CHN, le 02/10/2015 pour épistaxis et fièvre. A l'admission, l'examen avait noté un épistaxis, des ecchymoses au niveau du thorax et une fièvre non chiffrée. L'hémogramme montrait une anémie modéré (9,5g/dl), une hyperleucocytose à 15000/mm^3^, une thrombopénie à 90 000/mm^3^. Les bilans rénal et hépatique étaient perturbés. Le TDR pour le paludisme était négatif. Un prélèvement a été envoyé à l'INRSP pour la sérologie des fièvres hémorragiques. Les principaux résultats sont notés dans le Tableau 1. Un traitement symptomatique et antibiotique a été instauré. Au troisième jour de son hospitalisation, l’état général du patient s'est brusquement dégradé. Des douleurs thoraciques, une dyspnée au repos et une hématurie totale se sont installées. Le patient est décédé dans un état de choc hémorragique.

### Patient N° 4

GA, de sexe masculin, âgé de 17 ans, résidant à Mederdra/Trarza (Sud-Ouest du pays), sans antécédent notable, admis au service de médecine interne le 08/10/2015 pour un épistaxis, une hématémèse et fièvre à 39°C. L'hémogramme avait montré une anémie et une thrombopénie à 6600/mm^3^. Un prélèvement a été envoyé à l'INRSP pour la sérologie des fièvres hémorragiques et les principaux résultats sont notés dans le Tableau 1. Le patient avait bénéficié des transfusions sanguines (cinq concentrés plaquettaires), d'une antibiothérapie et d'un traitement symptomatique. L’évolution était favorable après cinq jours d'hospitalisation et le patient a été revu cinq jours plus tard en consultation externe.

### Patient N° 5

AE âgée de 29 ans, femme au foyer à 32 semaines d'aménorrhée, résidente à Djigueni/Hodh Charghi (Est du pays), sans antécédents particuliers admise au service de médecine interne du CHN le 1/10/2015 pour un épistaxis et une gingivorragie. L'examen physique à l'admission montrait un sub-ictère, des gingivorragies, un épistaxis et une obnubilation, une hyperthermie à 38°C dans un contexte d'altération de l’état général. L'hémogramme montrait une leucopénie à 3200, une anémie à 8,3 g/dl et une thrombopénie sévère à 7000/mm^3^. Le bilan hépato-rénal était perturbé. Les TDR pour le paludisme et l'AgHBs étaient négatifs. Un prélèvement a été envoyé à l'INRSP pour la sérologie des fièvres hémorragiques et les principaux résultats sont notés dans le Tableau 1. La patient avait bénéficié de transfusions sanguines (dix concentrés plaquettaires), d'une antibiothérapie et d'un traitement symptomatique. Un avortement spontané était observé au quatrième jour de son hospitalisation. Après une prise en charge, l’état clinique de la patiente s’était nettement amélioré. Elle était revue 10 jours plus tard à la consultation externe.

## Discussion

La FVR est une zoonose majeure, présente essentiellement sur le continent africain. Elle affecte essentiellement les ruminants domestiques et sauvages avec des conséquences désastreuses sur le cheptel africain [1], ce qui a des effets économiques dévastateurs sur les pays dont le commerce d'animaux constitue la principale source de revenu national [6]. La survenue des épizooties de FVR est souvent associée à des pluies diluviennes [1, 7, 8], ou à des modifications hydrographiques propices à la pullulation des moustiques compétents pour la FVR. En revanche, les conditions de maintien de ce virus entre deux épisodes de FVR restent mal connues [7, 8]. Il a été démontré pour certaines espèces de moustique du genre *Aèdes*, que le virus se maintient dans les œufs pendant de longues périodes, pouvant ainsi être transmis à la descendance [6–9]. Dans la majorité des cas, l'infection humaine à FVR est asymptomatique, mais elle peut également se manifester par un syndrome fébrile modéré non mortel. Néanmoins, certains patients peuvent développer un syndrome hémorragique et /ou des lésions neurologiques et oculaires [3, 4, 6, 8]. Ces complications sont variables: d'environ 20% pour le syndrome hémorragique, 1% pour les méningo-encéphalites et 0,3-2% pour les complications oculaires [3, 4, 8]. Nous rapportons, cinq cas de patients hospitalisés au service de médecine interne du CHN de Nouakchott (Mauritanie) durant le mois d'octobre 2015. Agés de 17 à 36 ans, quatre d'entre-eux étaient des hommes. Ils étaient originaires de différentes localités du pays, situées dans des zones endémiques pour le paludisme et présentaient tous un syndrome hémorragique fébrile. L'activité à risque d'infection retrouvée, était leur résidence en milieu rural. Des études antérieures menées dans le pays, avaient rapporté des cas de FVR au niveau des mêmes zones géographiques [7, 10]. Parmi nos patients, trois étaient des éleveurs et deux sans profession. A côté des agriculteurs et employés d'abattoirs, les éleveurs constituent une catégorie professionnelle à risque d'infection [1, 8]. Du fait de sa particularité de transmission à la fois directe et indirecte, voyager ou résider dans une zone épidémique, manipuler les viandes suspectes provenant d’ animaux malades ou être en contact avec les animaux malades confirmés ou non, constituent également un facteur à risque d'infection par le virus de la FVR [8]. La recherche des arbovirus par la technique « reverse transcriptase-polymerase chain reaction » (RT-PCR) a été effectuée au laboratoire de Virologie de l'INRSP. Elle était revenue positive pour le virus de la FVR et négative pour le virus du Crimée Congo, le virus de la fièvre jaune, le virus West Nile, le virus de la Dengue, le virus Chikungunya et le virus Zika. Cependant, malgré une prise en charge médicale correcte, nous avons déploré, trois décès. En effet, le syndrome hémorragique dont le traitement reste exclusivement symptomatique, représente la principale cause de la mortalité liée à la FVR [1].

## Conclusion

La FVR est une arbovirose dont les infections humaines sont le plus souvent asymptomatiques, mais en présence de symptômes, la pathologie peut engendrer des complications graves, parfois mortelles, d'où la nécessité d'une prise en charge médicale rapide et adéquate visant à réduire sa létalité importante. Compte tenu de la fréquence des épidémies en Mauritanie ces dernières années, un système d'alerte précoce impliquant, les services vétérinaires et sanitaires doit être réactivé pour anticiper les risques futurs d’épidémies et réduire le nombre de cas de FVR.
